# Components of action representations evoked when identifying manipulable objects

**DOI:** 10.3389/fnhum.2015.00042

**Published:** 2015-02-06

**Authors:** Daniel N. Bub, Michael E. J. Masson, Terry Lin

**Affiliations:** Department of Psychology, University of VictoriaVictoria, BC, Canada

**Keywords:** action representations, canonical and rotated view, object affordances, object identification, partial feature overlap

## Abstract

We examined the influence of holding planned hand actions in working memory on the time taken to visually identify objects with handles. Features of the hand actions and position of the object's handle were congruent or incongruent on two dimensions: alignment (left vs. right) and orientation (horizontal vs. vertical). When an object was depicted in an upright view, subjects were slower to name it when its handle was congruent with the planned hand actions on one dimension but incongruent on the other, relative to when the object handle and actions were congruent on both or neither dimension. This pattern is consistent with many other experiments demonstrating that a cost occurs when there is partial feature overlap between a planned action and a perceived target. An opposite pattern of results was obtained when the depicted object appeared in a 90° rotated view (e.g., a beer mug on its side), suggesting that the functional goal associated with the object (e.g., drinking from an upright beer mug) was taken into account during object perception and that this knowledge superseded the influence of the action afforded by the depicted view of the object. These results have implications for the relationship between object perception and action representations, and for the mechanisms that support the identification of rotated objects.

## Introduction

The functional properties of an object are an essential part of its conceptual representation; we understand what is meant by the phrase “a good pair of scissors” because we know that scissors are typically used for cutting and how reassuring it is when a pair cuts well. More contentious, though, is the role that function plays in the identification of visual objects. Neuroimaging studies have shown that identifying pictures of tools activates motor cortical regions (see Mahon and Caramazza, 2008, for a review), a result that has driven two widely held assumptions. First, it is claimed that to recognize the visual form of an object like a pair of scissors requires knowledge of its proper function. Second, the function of a tool is assumed to be represented in terms of the actual movements we produce and register when we interact with the object. For example, Martin et al. (2000) argued that “… information about object function needed to support tool recognition and naming is information about the patterns of visual motion and patterns of motor movements associated with the actual use of the object” (p. 1028).

Both of these claims are contentious. Apraxic patients are impaired in pantomiming the actions associated with a tool, and to a lesser extent, in the movements required to make use of the object itself. Yet they show relatively preserved understanding of the function of tools; for example, patients, despite their apraxia, are able to correctly judge that a scissors and a knife are used for similar purposes (see Garcea and Mahon, 2012, for a review). Clearly, knowing the general function of a tool includes a degree of abstraction beyond the movements associated with its use. There is also evidence that identifying human artifacts can occur purely on the basis of their shape, without regard to their function. Young children acquire the names of many such objects even before they have had the opportunity to learn about their functional properties (Merriman et al., 1993; Landau et al., 1998). Neuropsychological evidence further challenges the view that the ability to name tools depends on functional knowledge. Ochipa et al. (1989) documented the performance of a patient with ideational apraxia who could name tools despite showing severe impairment in tasks that assessed his understanding of their function (e.g., he failed to select a hammer as the correct tool when shown a piece of wood containing a partially embedded nail).

What then are we to make of the undeniable fact that identifying tools is associated with activity in motor cortical regions? Although this result in itself does not necessarily imply a causal role for motor representations in perception (see Mahon and Caramazza, 2008), enough additional evidence has accumulated, some of which we review below, to suggest that motor representations do exert an influence—yet to be adequately defined—on the perception of manipulable objects. In what follows, we develop an experimental approach that sheds light on the motor features influencing the perception of handled objects like beer mugs and frying pans. Our research builds on previous work establishing that secondary tasks that require the programming of hand actions have an adverse impact on the ability of normal subjects to identify tools and other graspable objects.

## Actions play a role in object identification

Witt et al. (2010) required participants to squeeze a small foam ball with their right or left hand while identifying pictures of tools or animals. Naming was delayed when pictures of tools were displayed with their handles aligned toward the hand carrying out the squeezing task. No comparable effect was obtained for depicted animals presented with their heads oriented toward or away from the responding hand. The authors suggested that squeezing a ball engages motor processes that are also needed to evoke a left or right-handed action associated with grasping the depicted tool. Presumably, these motor representations are causally implicated in the naming task.

More recently Yee et al. (2013) documented the effect of a secondary motor task on the perceptual identification of objects. Participants carried out a three-step sequence of meaningless actions using both hands while concurrently identifying objects associated with a high or low degree of motor experience. A block of trials performed without concurrent motor demands served as a baseline condition. Naming accuracy for objects rated as being frequently touched (e.g., toothbrush) showed greater interference from the motor task than objects (like a bookcase) associated with fewer motor memories. The authors inferred, given these results, that motor information is part of the representation used when identifying manually experienced objects.

An interesting set of methodological issues emerges if we compare the logic of the two studies we have just summarized. The approach favored by Yee et al. (2013) relies on the claim that object concepts in long-term memory are abstracted away from specific instances. The procedure they used generated its effects not because of any degree of similarity between the actions involved in the secondary task and the actions associated with the target objects. Rather, the secondary task presumably demanded motor resources that were also needed for the identification of objects typically associated with a high degree of manipulability. The rival assumption tacitly made by Witt et al. (2010) is that access to the conceptual identity of an object can never be completely separated from its visible form; motor interference depends on the spatial overlap between the left/right hand carrying out the secondary task and a left or right handed grasp evoked by a tool. Indeed, we believe this assumption must surely be valid at some level; the token form of a beer mug (say, rotated with the handle facing upwards) is after all an entry point to the conceptual representation of beer mugs in general. Thus, we are sympathetic to the idea that actions afforded by the handle of an object in a particular orientation play some role in processing its conceptual identity. Nevertheless, it is also true that an object concept is generally founded on a type rather than a specific token identity, consistent with the opposing standpoint taken by Yee et al., As such, actions that are implicated in object perception surely cannot be based entirely on a particular depicted form. How to reconcile these discrepant alternatives?

## Motor features in object naming

In this section, we describe the logic of our approach to the question we have just posed, which draws on a large body of previous research documenting that a prepared action maintained over a short duration can disrupt performance on an intervening perceptual task (e.g., Hommel et al., 2001; Hommel, 2009). This widely obtained result is taken as support for the claim that action and perception share common representational substrates; a motor task that requires the maintenance of features in working memory will interfere with a perceptual task that invokes the same features. The particular pattern of interference effects is surprising but has nonetheless been repeatedly observed. Performance is impaired only when there is a partial match between the constituents of the working memory task and the perceptual task. A complete match or total mismatch of features has no effect on perception. Hommel (2004) pointed out that this outcome implies not so much a benefit in repeating a feature conjunction as a cost incurred when there are features partially shared between different perceptual-motor events. A single recurring feature in perception will trigger retrieval of a previous event in working memory by spreading activation, and the ensuing conflict, brought about by a mismatching feature or set of features, will hamper stimulus identification and/or response selection (for additional theoretical details, see Stoet and Hommel, 2002; Hommel et al., 2004).

Experiments on motor-visual interference generally incorporate abstract symbols as objects and arbitrary responses as actions, to facilitate parametric variation of elementary features like spatial orientation and position. Nevertheless, given certain assumptions (see below), it is possible to apply the same basic principles underlying the pattern of effects we have just described to the more realistic world of everyday manipulable artifacts and their associated motor representations.

What kind of motor features are evoked by an upright beer mug with its handle on the right? The action corresponding to this depicted view of the object is a right handed, closed grasp, with the wrist oriented vertically (i.e., the ventral and dorsal surfaces of the wrist are vertically perpendicular to the ground). By contrast, a frying pan with the handle on the left requires a left-handed closed grasp with the wrist oriented horizontally (i.e., the wrist is pronated so that its ventral and dorsal surfaces are parallel to the ground). Thus, we can reasonably conjecture that features such as hand (left vs. right) and wrist orientation (vertical vs. horizontal) would be recruited as part of the motor representations that are implicated in the identification of handled objects. A test of this conjecture, based on motor interference effects generated by a secondary task, is relatively straightforward. We arranged matters so that the constituents of a prepared set of actions maintained in working memory incorporated the above two features, and we examined the impact of this secondary task on the time taken to perceptually identify pictures of handled objects (Bub et al., 2013). Remarkably, our results fully replicated the pattern of interference effects typically obtained with abstract symbols as objects and arbitrary stimulus-response mappings. Object naming latency was slowed when a single motor feature was shared between the prepared action (left or right handed action; vertical or horizontal grasp posture) and the affordance of the target object. Latencies were faster (and accuracy was higher) when the planned action and perceived object shared both or neither of these features. Thus, the manipulability of an object can be decomposed into constituent features that are part of its semantic representation. A particular strength of our methodological approach is that it promises to further clarify the computational role of motor features in the perceptual classification of everyday manipulable objects.

The objects in Bub et al. (2013) were all upright and so, apart from the fact that they varied with respect to the left/right positioning and vertical/horizontal orientation of their handles, each object's depicted view matched its canonical view. We cannot therefore answer the fundamental question we posed earlier: what is the relative contribution of the actions associated with the depicted and canonical form to the identification of a manipulable object? To clarify this issue, we need to distinguish the actions associated with the upright canonical description of an object from those evoked by its depicted form. Imagine a beer mug on its side with the handle facing upwards. The motor features activated as part of the conceptual identity of the object would reference the grasp associated with its upright, canonical form. Thus, a vertical rather than a horizontal wrist orientation should be invoked, while the reverse would be the case for the depicted view. We can determine which of these parameters of the wrist orientation feature is recruited for identification by examining the pattern of interference effects generated by planned actions held in working memory. A motor feature shared between the constituents of working memory and the actions recruited by the object will have an adverse impact on naming performance. In the case of the rotated beer mug, does the shared feature correspond to a vertical wrist orientation (matching the canonical form of the object) or a horizontal wrist orientation (conforming to the depicted view)?

What of the motor feature corresponding to the left/right choice of hand? The canonical description might include the fact that we typically use our dominant hand to lift and use a manipulable object. However, a more complex possibility should be considered. As we have noted, the depicted view of an object is the entry point to knowledge of its identity. Assume that naming an object depends in part on translating the rotated form of an object into a canonical upright representation. An object like a beer mug will evoke a left- or right-handed grasp depending on the location of the handle after rotation. For example, a horizontal beer mug with the mouth or opening on the right will yield a left-handed grasp when rotated into an upright position. In general, the token form of an object may determine whether the canonical representation activates a left- or right- hand grasp if motor features are consulted as part of the naming process.

To summarize, we conjecture that the speeded naming of manipulable objects (tools and utensils) should recruit the motor features left/right hand, and vertical/horizontal wrist orientation. We will rely on the pattern of interference produced by a secondary working memory task incorporating these features to clarify the nature of the motor representations contributing to performance.

## Experiment 1

We investigated the influence of action features held in working memory on the identification of pictured objects presented either in their canonical view or rotated 90° so that the object's handle was shifted from a horizontal to a vertical orientation, or vice versa. The critical question was whether under this rotation the object would be encoded in its depicted view or in its canonical view and, more particularly, how that encoding would interact with the action representations held in working memory. One possibility is that the relation between the features of the hand actions held in working memory and the depicted features of the object's handle would determine congruency and thereby the pattern of response times for partial feature overlap, complete overlap, and no overlap conditions. Alternatively, congruency might be driven by the relation between the features of the hand actions and the canonical features of the object's handle, not its depicted features. Testing rotated views of the objects allowed us to address this issue.

As an additional test of the nature of the encoding of rotated objects, we included a set of objects that do not have a standard canonical view, inasmuch as they are very often seen and used both in a horizontal and in a vertical position (e.g., hair brush, wrench). For these *acanonical* objects, we anticipated that the influence of working memory load would be determined by the depicted view of the object because there would be no strong canonical view to oppose it.

## Methods

### Subjects

Thirty students at the University of Victoria participated to earn extra credit in an undergraduate psychology course. The experiments reported here were approved by the University of Victoria Human Research Ethics Committee.

### Materials

**Figure 1:**
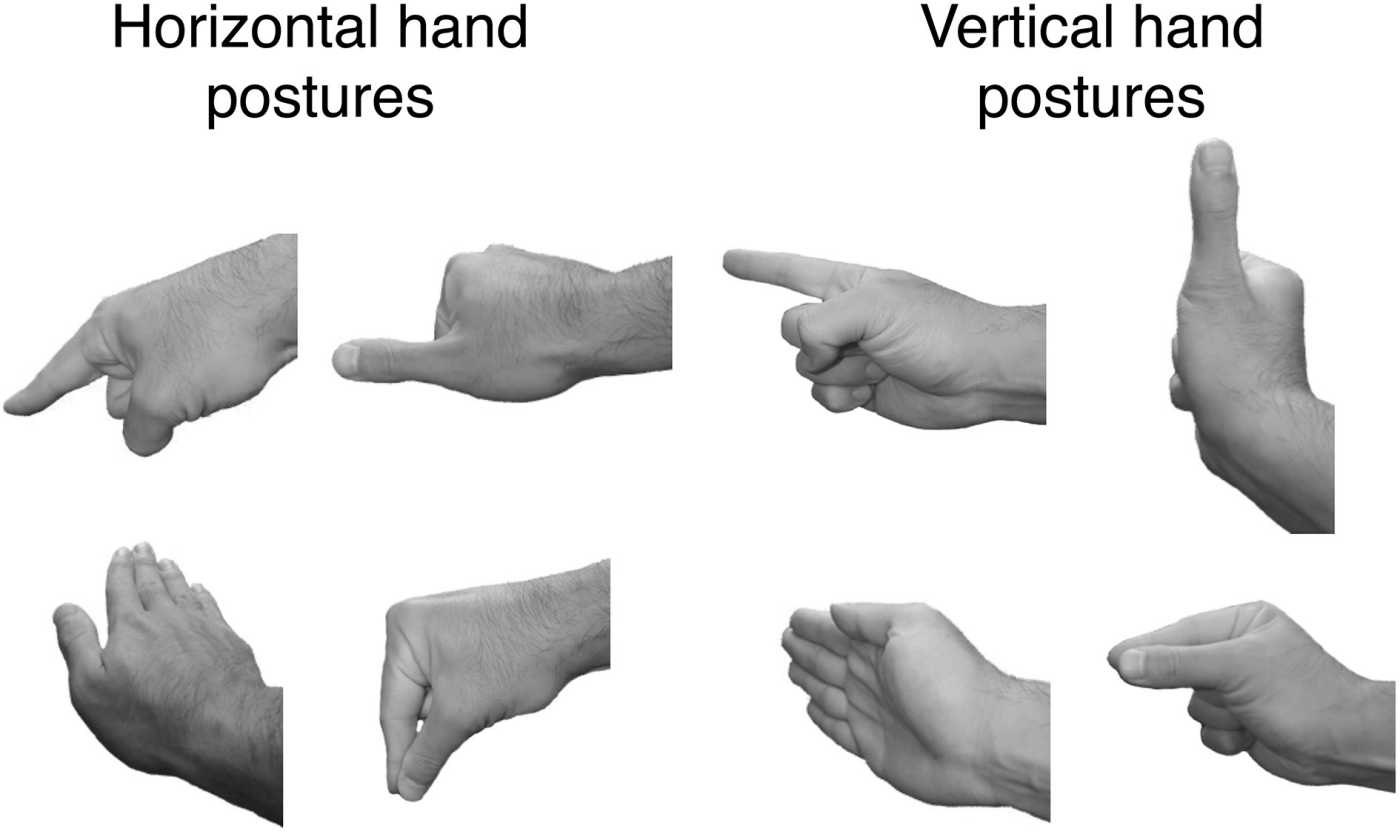
**The four hand postures used for defining the working memory load on critical trials**. Each posture was formed with the wrist in a horizontal position (left side of figure) and in a vertical position (right side of figure). Two postures were selected (both horizontal or both vertical) as the working memory load for a trial.

Four hand postures, distinct from a simple power grasp^1^, were selected for use as memory load stimuli. The four postures were: extended forefinger, extended thumb, flat palm, and precision grip with thumb and forefinger. A grayscale digital photograph was taken of a male right hand formed in each of these postures with the wrist oriented horizontally (so the palm of the hand faced downward) and again with the wrist rotated vertically (i.e., the wrist continued to be parallel to the ground, but its dorsal and ventral surfaces were now oriented vertically; see Figure 1). Each of these eight photographs was rendered in a left-handed pose by creating a mirror image reflection of the original image.

**Figure 2:**
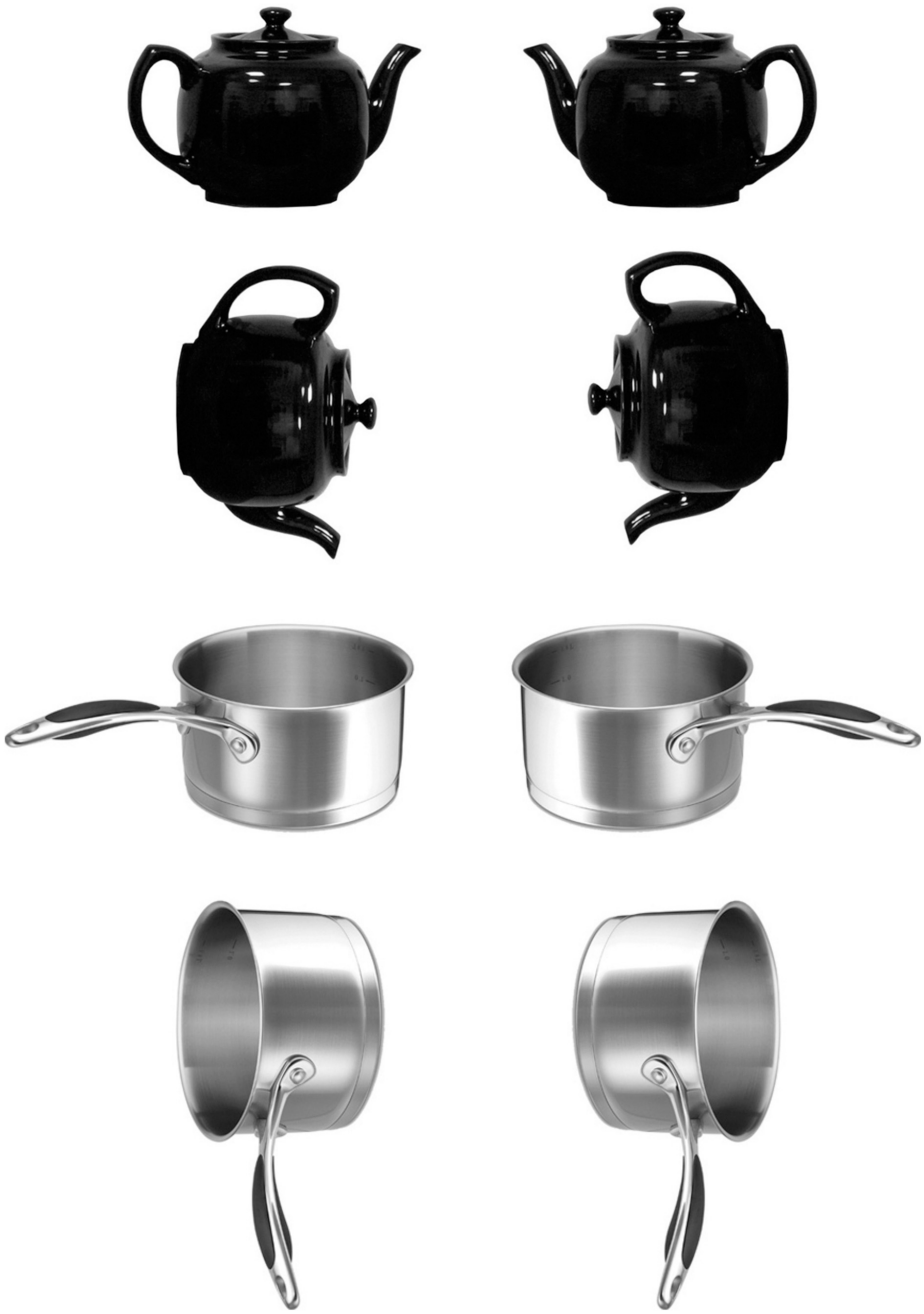
**Examples of two objects (one with a vertical handle when upright and one with a horizontal handle when upright) shown in each of the four possible views**. Images in the left column represent objects that are most readily grasped with the left hand, and images in the right column show objects that ought to be grasped with the right hand.

Twenty-four object types were chosen for use as target objects. All were handled objects that are typically used by applying a power grasp to the object's handle. Eight of the object types had a handle that is vertically oriented when the object is in its canonical position (e.g., beer mug), eight were objects that have a horizontally oriented handle (e.g., frying pan) when in their canonical position, and eight were acanonical objects (often experienced with their handles in either orientation). A list of the names of the 24 object types is given in the Supplementary Material. Four token images of each type were chosen from various internet sites (e.g., four different knives), yielding 96 token images. Each of the 96 token images were rendered as grayscale digital images providing a profile view of the object (see Figure 2). Two variants of each image were created, one with the handle facing to the right (inviting a right-hand grasp) and one with the handle facing to the left.

A rotated view of the right- and left-hand variant of each token image was created by rotating the image 90° such that a canonical object with a vertical handle now had its handle oriented horizontally and positioned on the upper part of the image. For objects with horizontal handles, the chosen 90° rotation caused the handle to point downward. For acanonical objects, we arbitrarily deemed images with the handle in a vertical orientation to be upright, and images with a horizontally oriented handle to be rotated. Figure 2 shows examples of the upright and rotated images for two objects, one whose canonical handle orientation is vertical and the other horizontal. Note that for both the upright and rotated views, a depicted image invites a grasp by one or the other hand. In the case of the canonical view, the handle is positioned to favor one hand. In the rotated view, the preferred hand is determined by the principle of commensurability (Masson et al., 2011), whereby the choice of hand for grasping a rotated object is determined by whether using a particular hand will allow the object to be brought into its upright, functional position with a comfortable wrist rotation (see also Rosenbaum et al., 1990). For example, consider the image of the rotated teapot on the left side of Figure 2. Grasping an object oriented that way with the left hand, then rotating the wrist counterclockwise 90° would lead to an upright teapot in a comfortable position. Using the right hand to grasp that object, however, would require an awkward and uncomfortable wrist rotation to bring the object to an upright position.

### Design

On each critical trial of the experiment, subjects were presented two hand actions (represented by images of hand postures as in Figure 1) as a working memory load. These two actions involved the same hand (right or left) and the same wrist orientation (horizontal or vertical), but differed in hand posture. The primary manipulation in the experiment was the relationship between the hand and orientation of the two actions in working memory and the right/left alignment and the orientation of the handle of the object to be named on that trial. We use the term alignment to refer to the congruency between the hand actions and the object with respect to the hand used for the actions and the side favored by the handle. For example, actions using the right hand are congruent with an object whose handle is on the right side of the object's image or, in the case of a rotated object, for which a right handed grasp would be commensurate with its function. Orientation refers to the congruency between the wrist orientation of the hand actions in working memory and the orientation of the target object's handle. For example, hand actions with a horizontally oriented wrist posture are congruent with an image of an upright sauce pan.

There were 16 conditions in the experiment, defined by the alignment and orientation of the object's handle and the alignment and orientation of the hand actions that formed the working memory load. Three blocks of 96 critical trials were presented, yielding a total of 288 critical trials. Each of the 96 token images was presented once in each block. Within each block, six objects (two of each class: horizontal, vertical, and acanonical) were randomly assigned to each of the 16 conditions. The assignment of objects to conditions varied across subjects so that each object type was tested equally often in each condition. The specific object image that was presented depended on the condition to which the object was assigned. For example, if an object with a vertical handle when in its upright position were assigned to the condition with a horizontal handle and right alignment, then the rotated image of the object, with its top to the left and its bottom to the right, was used (e.g., the lower right image of the teapot in Figure 2). The four hand postures were arranged into six different pairs. Each pair was used with one of the objects in each of the 16 conditions in a block of trials. The order of presentation of the two hand postures within a trial was randomly determined.

### Procedure

All images of hands and objects were scaled to fit within a square extending 14.5° of visual angle on each side when viewed from 50 cm. Images were displayed on an LCD monitor controlled by a Macintosh desktop computer. Subjects were tested individually in a quiet room under the supervision of an experimenter who provided instructions and scored responses as they occurred. Subjects wore a headset with a microphone to detect their vocal responses.

**Figure 3:**
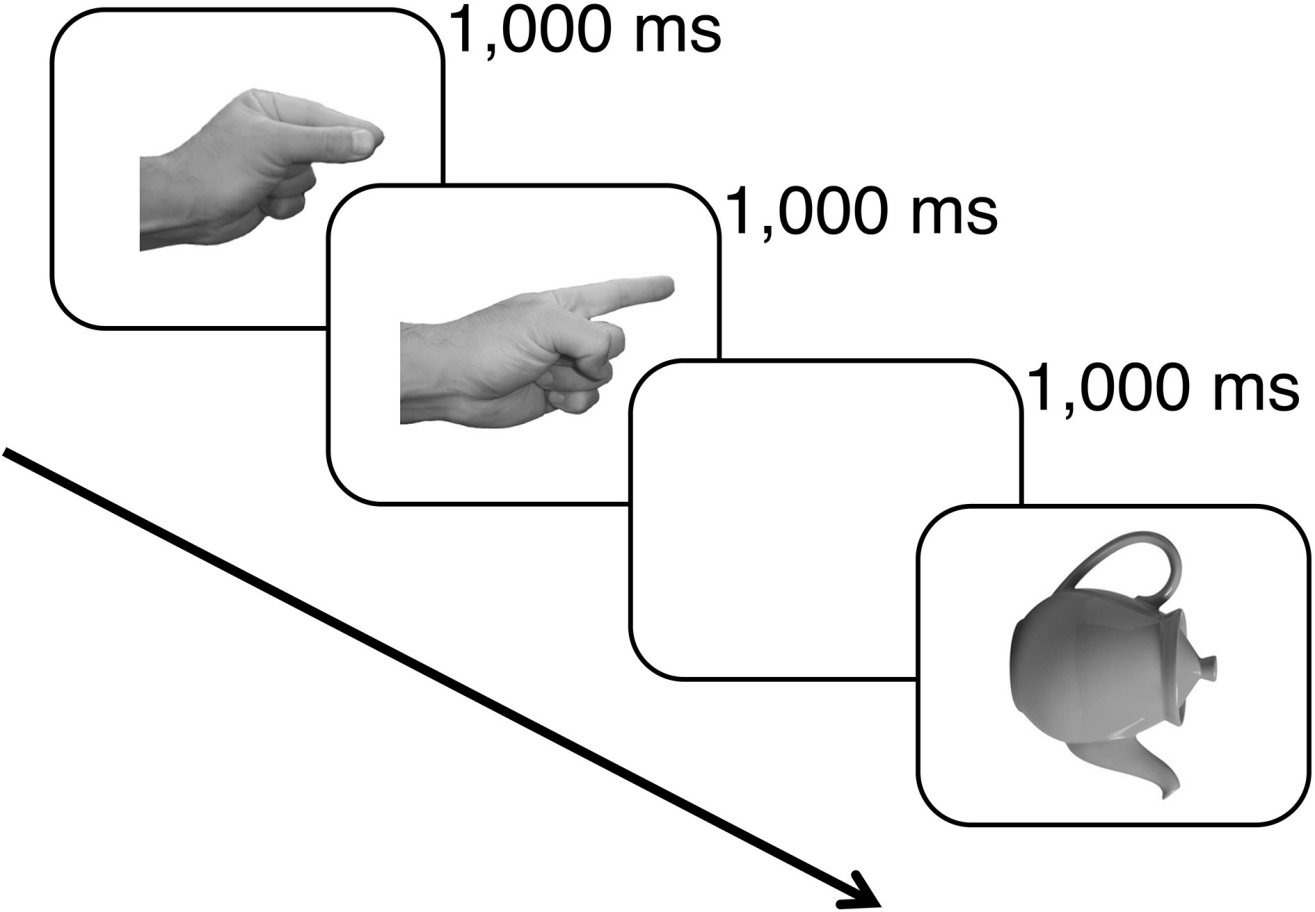
**An example of the events presented on a critical trial**. Each hand action cue was shown for 1000 ms followed by a 1000-ms blank screen and then the picture of the target object that was to be named. The object remained in view until a vocal response was detected.

In the first phase of the experiment, subjects were familiarized with the set of hand actions and their associated cues. They were given an opportunity to pantomime each combination of hand shape and wrist orientation with each hand in response to the pictured hand cues. Subjects were also given practice at naming the left-facing upright images of each of the object tokens. In the second phase of the experiment, subjects were presented 288 critical trials. On each trial subjects were shown for 1000 ms each of the cues for the two hand actions that constituted the working memory load for that trial, followed by a 1000-ms blank display. The pictured object then appeared and subjects were instructed to name the object as quickly and accurately as possible (see Figure 3). Their vocal responses were detected by the microphone on the headset they wore, and the experimenter pressed a key on the computer keyboard to score the accuracy of the response. On a randomly selected 25% of trials, after the vocal response a signal appeared on the monitor indicating that the subject was to pantomime the two hand actions that were held in working memory on that trial. This task ensured that subjects attended to and maintained in memory the hand actions presented on each trial.

## Results and discussion

### Report of hand actions

When reporting the hand actions held in working memory, subjects were scored correct if they reported both actions, regardless of the order in which they were reported. The mean percent correct was 79.3%. This level of performance indicates that the working memory task was a demanding one, but that subjects were able to maintain the assigned actions in most trials (the lowest scoring subject was correct on 70.4% of the trials).

### Statistical analyses

The analyses we report provide both the outcome of a null hypothesis significance test and the corresponding Bayes factor (BF) generated using the BayesFactor package in the open source statistical program R, described by Rouder et al. (2012). The Bayes factor we report for an effect indicates the ratio of the strength of evidence supporting a model of the data that includes all effects in the design relative to a model that excludes only the effect of interest. Larger values of the Bayes factor indicate stronger evidence for the effect.

Naming latencies for correct responses were included in the analyses if they were longer than 200 ms and shorter than 2600 ms. The lower bound was intended to eliminate extraneous activations of the microphone and the upper bound was selected so that no more than 0.5% of the longest response times were removed as outliers (Ulrich and Miller, 1994).

In the analyses, we were interested in congruency between the object to be named and the actions held in working memory with respect to two attributes: hand alignment and wrist orientation. The conditions we used constituted a factorial manipulation of these two types of congruency. For upright object images, congruency was determined in the obvious way (e.g., left-hand actions were congruent with an object pictured with its handle on the left; vertical wrist orientation in hand actions was congruent with an object whose handle is vertically oriented, such as teapot). For rotated object images, congruency of alignment was determined by which hand would be commensurate with grasping the object and comfortably rotating the wrist to bring it to an upright position. Consider, for instance, the sauce pan in the bottom right of Figure 2. Its handle would be considered to be aligned with the right hand because a grasp made with that hand could be followed by a 90° wrist rotation to bring the pan into a functional position. Congruency of orientation for rotated images was determined by the depicted view of the object. For a rotated beer mug, for example, a horizontal action was deemed congruent.

### Acanonical objects

Analysis of object naming performance was conducted separately for the acanonical objects on one hand, and for the horizontal and vertical objects on the other hand. It was expected that because acanonical objects lacked a typical horizontal/vertical view, they would interact with the actions held in working memory differently than would objects characterized by a typical upright view.

**Figure 4:**
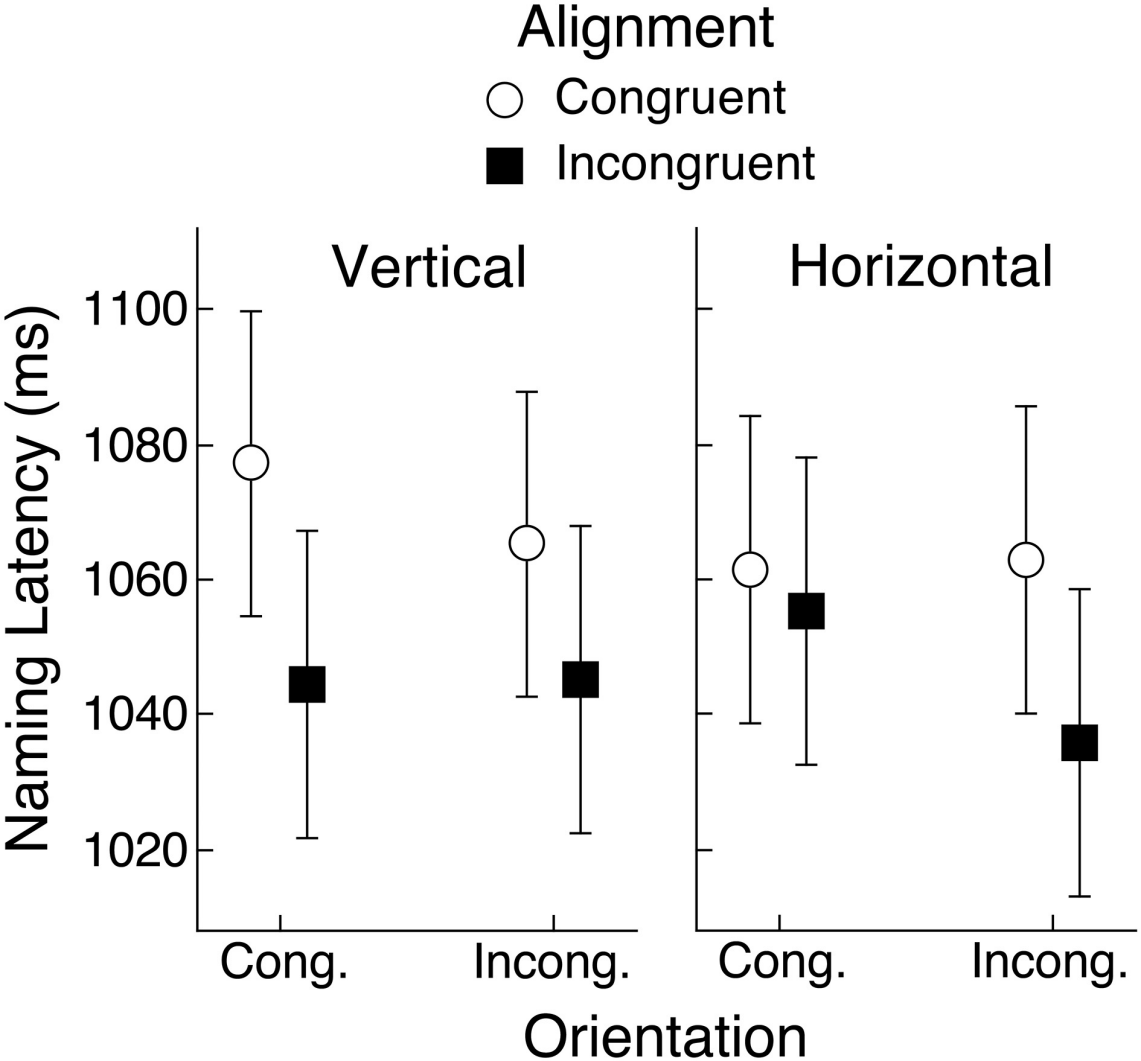
**Mean object naming latency for acanonical objects in Experiment 1 as a function of alignment and orientation congruency with respect to hand actions held in working memory**. Upright and rotated views of these objects were arbitrarily defined by vertical and horizontal orientation of their handles, respectively. Error bars are 95% within-subject confidence intervals (Loftus and Masson, 1994; Masson and Loftus, 2003).

Mean naming latencies for acanonical objects are shown in Figure 4, representing conditions defined by object view (horizontal or vertical), congruency of the orientation of the hand actions held in working memory relative to the viewed object (congruent or incongruent), and congruency of the left-right alignment of the hand actions held in working memory and the viewed object (congruent or incongruent). For example, a toothbrush presented in a horizontal orientation with its head on the left and its handle pointing to the right, would be congruent on orientation and alignment with hand actions using the right hand with a horizontally oriented wrist, but incongruent on both dimensions with hand actions using the left hand with a vertically oriented wrist.

A repeated-measures analysis of variance (ANOVA) with object view, orientation, and alignment as factors produced only a main effect of alignment, *F*_(1, 29)_ = 10.77, *MSE* = 2574, *p* < 0.01, *BF* = 4.5. For all other effects, *F*s < 1 (*BF*s < 0.4). As can be seen in Figure 4, naming latencies were longer when the hand actions in working memory and the object handle were congruently rather than incongruently aligned (1067 vs. 1045 ms). Note that the lack of an effect of object view is consistent with our assumption that this set of objects is frequently experienced in both horizontal and vertical orientations. The mean naming error rate was 0.6% and across 240 cells of the design (30 subjects × 8 conditions), only 16 had any errors. Therefore, no inferential analysis was applied to the error data.

For acanonical objects, the dimension of orientation congruency did not influence naming time, unlike our previous results (Bub et al., 2013), in which naming time was sensitive to the combination of alignment and orientation congruency. This result suggests that subjects were sensitive not only to the depicted view of the object (as clearly indicated by the effect of alignment), but also to prior knowledge and experience, whereby this set of objects would frequently have been encountered in both horizontal and vertical views. The interference effect associated with congruent hand alignment could be attributed to the binding of that feature with the action plan held in working memory, making it unavailable to processes responsible for identifying the target handled object (Hommel et al., 2001; Hommel, 2004). The unavailability of the orientation feature, which is assumed to be bound to the hand actions in working memory, apparently could be compensated for by knowledge of prior experience with the target object positioned in a manner opposite to the depicted view. As a result, congruency of the orientation feature had no influence on object naming.

### Objects with a canonical view

The mean naming error rate was 1.5% and an ANOVA computed with object view and congruency for alignment and orientation found no significant effects.

**Figure 5:**
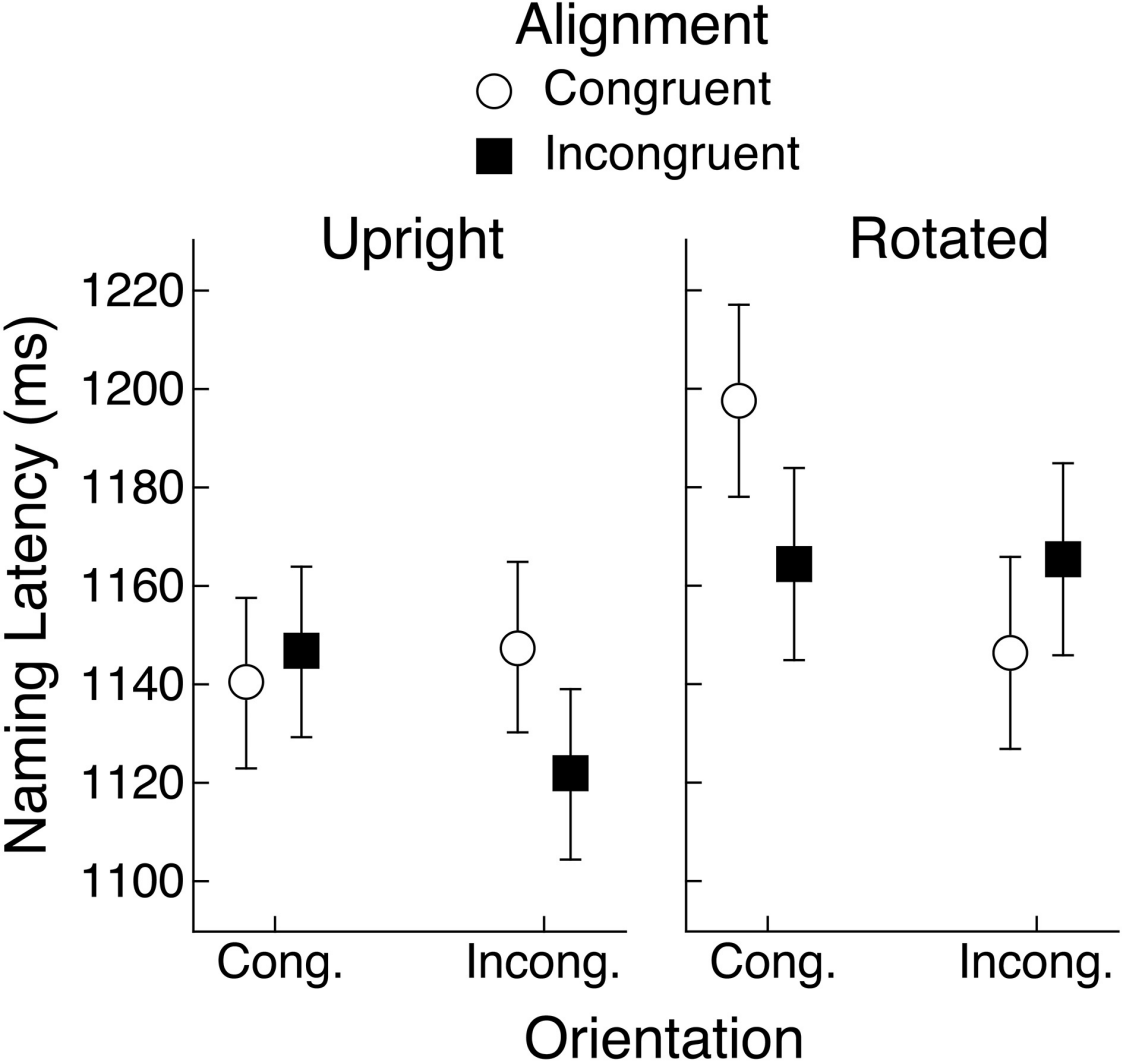
**Mean object naming latency in Experiment 1 for upright and rotated views of objects having a canonical view**. Means are shown for the four conditions defined by congruency of alignment and orientation between the object's handle and the hand actions held in working memory. Error bars are 95% within-subject confidence intervals.

The mean naming latencies for objects that have a strong, typical view are shown in Figure 5. An ANOVA applied to the latency data with object view and congruency for alignment and orientation as repeated-measures factors revealed a main effect of object view, *F*_(1, 29)_ = 11.40, *MSE* = 4476, *p* < 0.01, *BF* = 627.9, whereby objects were named faster if they were presented in their upright rather than rotated view (1139 vs. 1168 ms). The only other significant effect was the three-way interaction between object view (upright, rotated), alignment congruency, and orientation congruency, *F*_(1, 29)_ = 14.97, *MSE* = 1768, *p* < 0.01, *BF* = 13.6. This interaction is consistent with what would be expected if rotated objects were encoded so that action representations associated with their canonical view were evoked, rather than actions implied by their depicted view.

To follow up the three-way interaction, we conducted separate ANOVAs for upright and rotated objects. For upright canonical objects, we had expected to replicate the pattern of congruency effects reported by Bub et al. (2013) and also found in a replication study in our lab (Bai, 2013), whereby shorter naming latencies were obtained when both alignment and orientation were congruent or both were incongruent, relative to when one dimension was congruent and the other incongruent. Figure 5 indicates that this pattern was only partly replicated, given that a very weak orientation effect was found when alignment was congruent. An ANOVA applied only to upright objects with alignment and orientation congruency as repeated-measures factors found a significant interaction, but the Bayesian analysis provided virtually no support for it, *F*_(1, 29)_ = 4.58, *MSE* = 1670, *p* < 0.05, *BF* = 1.1. Neither main effect was significant.

For rotated objects, an ANOVA with alignment and orientation congruency as repeated-measures factors yielded a significant interaction that was also supported by the Bayesian analysis, *F*_(1, 29)_ = 5.74, *MSE* = 3547, *p* < 0.05, *BF* = 4.9. In addition, there was a main effect of orientation congruency, *F*_(1, 29)_ = 6.19, *MSE* = 3.074, *p* < 0.05, *BF* = 3.5, but not of alignment congruency. If rotated objects had been encoded purely on the basis of their depicted view, then we should have seen an interaction between alignment and orientation congruency much like that observed by Bub et al. (2013). Instead, however, the pattern shown in Figure 5 is more in keeping with the one-feature overlap interference effect that would occur if subjects had encoded the canonical features of objects, rather than their depicted features. That is, response time was particularly long if both alignment and depicted handle orientation were congruent with the hand actions held in working memory. But if we suppose the canonical view of the target object had been encoded, then what is labeled as congruent orientation in Figure 5 becomes incongruent, and vice versa, so that we now have a pattern that more closely resembles that reported by Bub et al. When alignment was incongruent, however, the effect of orientation congruency was virtually non-existent. Therefore, neither the upright nor the rotated views produced a pattern of congruency effects that fully matches that obtained by Bub et al. Consequently, we are not yet in a position to draw strong conclusions about how subjects encoded objects presented in a rotated view.

## Experiment 2

It is possible that the congruency effects found in Experiment 1 for objects that have a typical view were modulated by the inclusion of acanonical objects in the set of target objects. Indeed, Bub et al. (2013) did not include acanonical objects in their experiment when demonstrating the partial overlap interference effect. Therefore, in Experiment 2 we replicated Experiment 1 but excluded acanonical objects. In addition, we examined the response time distributions in Experiment 1 and observed that the partial overlap interference effect was most apparent among the lower two thirds of response times. In a effort to maximize the effect in Experiment 2, we encouraged subjects to make faster responses by providing them with only a brief view of a target object (150 ms) followed by a visual mask.

## Methods

### Subjects

Thirty subjects were recruited from the same source as in Experiment 1, although none had participated in that experiment.

### Materials and design

The same images of hand postures and objects were used as in Experiment 1, except that the acanonical objects were excluded. The remaining 64 objects were each presented once in each of four successive blocks, producing a total of 256 critical trials. Within each block, objects and hand actions were again assigned to the same 16 conditions as in Experiment 1 and these assignments varied across subjects so that each object concept was tested equally often in each condition.

### Procedure

The procedure was the same as in Experiment 1, except that on critical trials, the target object was in view for only 150 ms before being replaced by a pattern mask.

## Results and discussion

**Figure 6:**
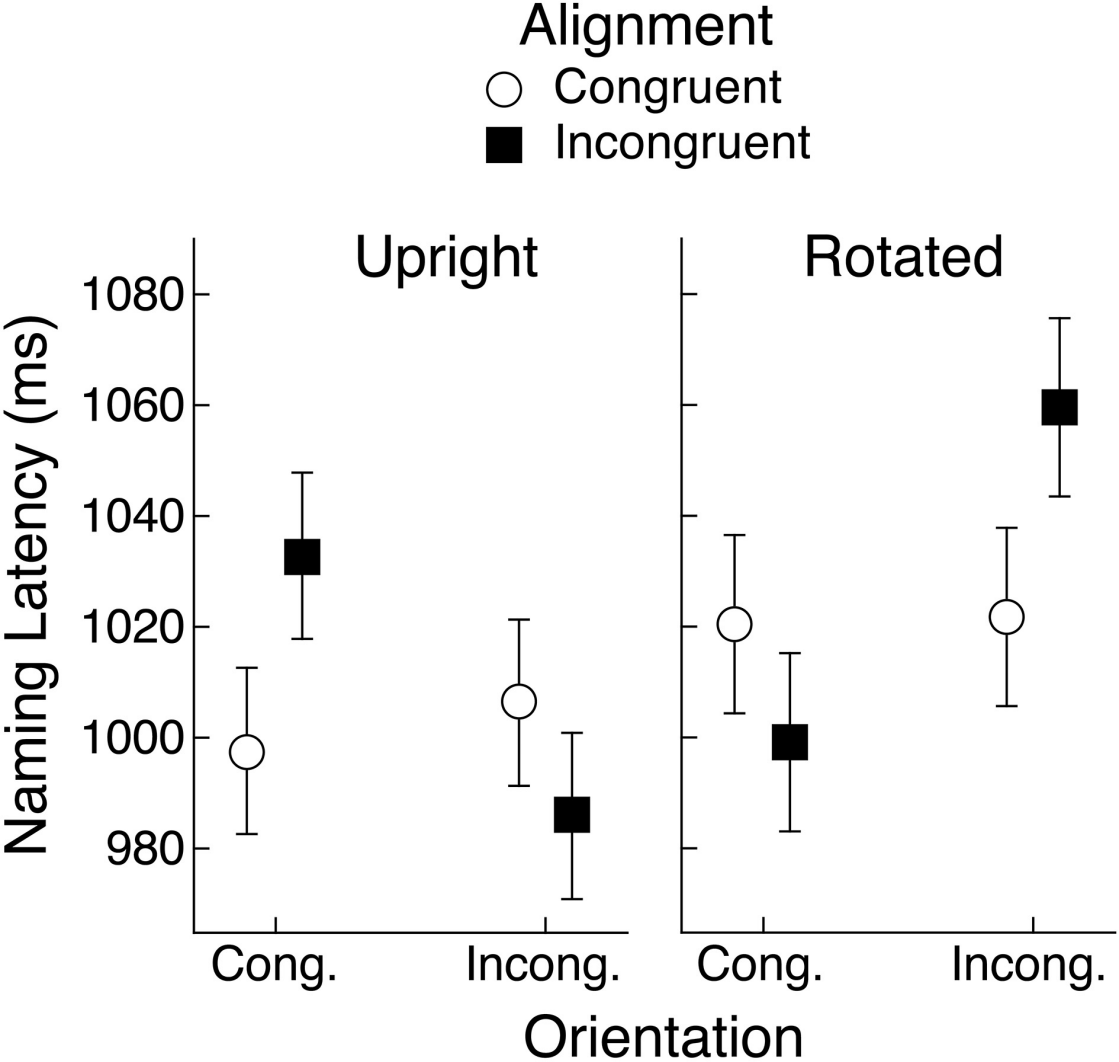
**Mean object naming latency in Experiment 2 for upright and rotated views of objects having a canonical view**. Means are shown for the four conditions defined by congruency of alignment and orientation between the object's handle and the hand actions held in working memory. Error bars are 95% within-subject confidence intervals.

Subjects correctly reported the hand actions that were held in working memory on an average of 79.4% of the trials on which they were probed to report them. As in Experiment 1, naming latencies less than 200 ms were excluded from analysis, as well as latencies in excess of 2800 ms. The upper cutoff was set so that fewer than 0.5% of correct trials were excluded. The mean naming latencies are shown in Figure 6. An ANOVA with object view (upright vs. rotated) and alignment and orientation congruency as repeated-measures factors indicated that upright objects were named faster than rotated objects (1006 vs. 1025 ms), *F*_(1, 29)_ = 8.64, *MSE* = 2660, *p* < 0.01, *BF* = 30.9. There was also a significant interaction between object rotation and orientation congruency, *F*_(1, 29)_ = 29.08, *MSE* = 1256, *p* < 0.01, *BF* = 878.7, but this effect was superseded by the significant three-way interaction, *F*_(1, 29)_ = 21.44, *MSE* = 2309, *p* < 0.01, *BF* > 1000. No other factors were significant.

### Upright objects

The three-way interaction was examined by computing separate ANOVAs for each object rotation condition with alignment and orientation congruency as repeated-measures factors, as in Experiment 1. For upright objects, there was a main effect of orientation congruency, with longer latencies when the object handle and the hand actions had congruent orientations rather than incongruent orientations (1015 vs. 996 ms), *F*_(1, 29)_ = 6.31, *MSE* = 1679, *p* < 0.05, *BF* = 2.8. But there was also a significant interaction between alignment and orientation congruency, *F*_(1, 29)_ = 15.96, *MSE* = 1459, *p* < 0.01, *BF* = 70.5. This interaction generally conforms to the pattern reported by Bub et al. (2013), although the effect of orientation congruency was rather weak when alignment was congruent. This was also the case in Experiment 1.

### Rotated objects

For rotated objects, the ANOVA with alignment and orientation congruency as factors yielded a main effect of orientation congruency, although here latencies were shorter in the congruent case (1010 vs. 1041 ms), *F*_(1, 29)_ = 16.04, *MSE* = 1746, *p* < 0.01, *BF* = 82.4. Note that if we assume, as suggested above, that subjects encode rotated objects in their canonical view, then the orientation congruency effect can be seen as an interference effect [longer latencies when the encoded (canonical) orientation of the object's handle matches the orientation of hand actions held in working memory], just as was seen with upright objects. The alignment by orientation congruency interaction was also significant, *F*_(1, 29)_ = 10.43, *MSE* = 2517, *p* < 0.01, *BF* = 62.4. As in Experiment 1, the pattern of means is similar to what would be expected from the Bub et al. (2013) findings if we assume that rotated objects were encoded in their canonical view and that it was this view that determined congruency with the orientation of hand actions held in working memory. Once again, however, the fit is not perfect because in this case the orientation congruency effect was rather weak when alignment was congruent.

### Error rates

**Figure 7:**
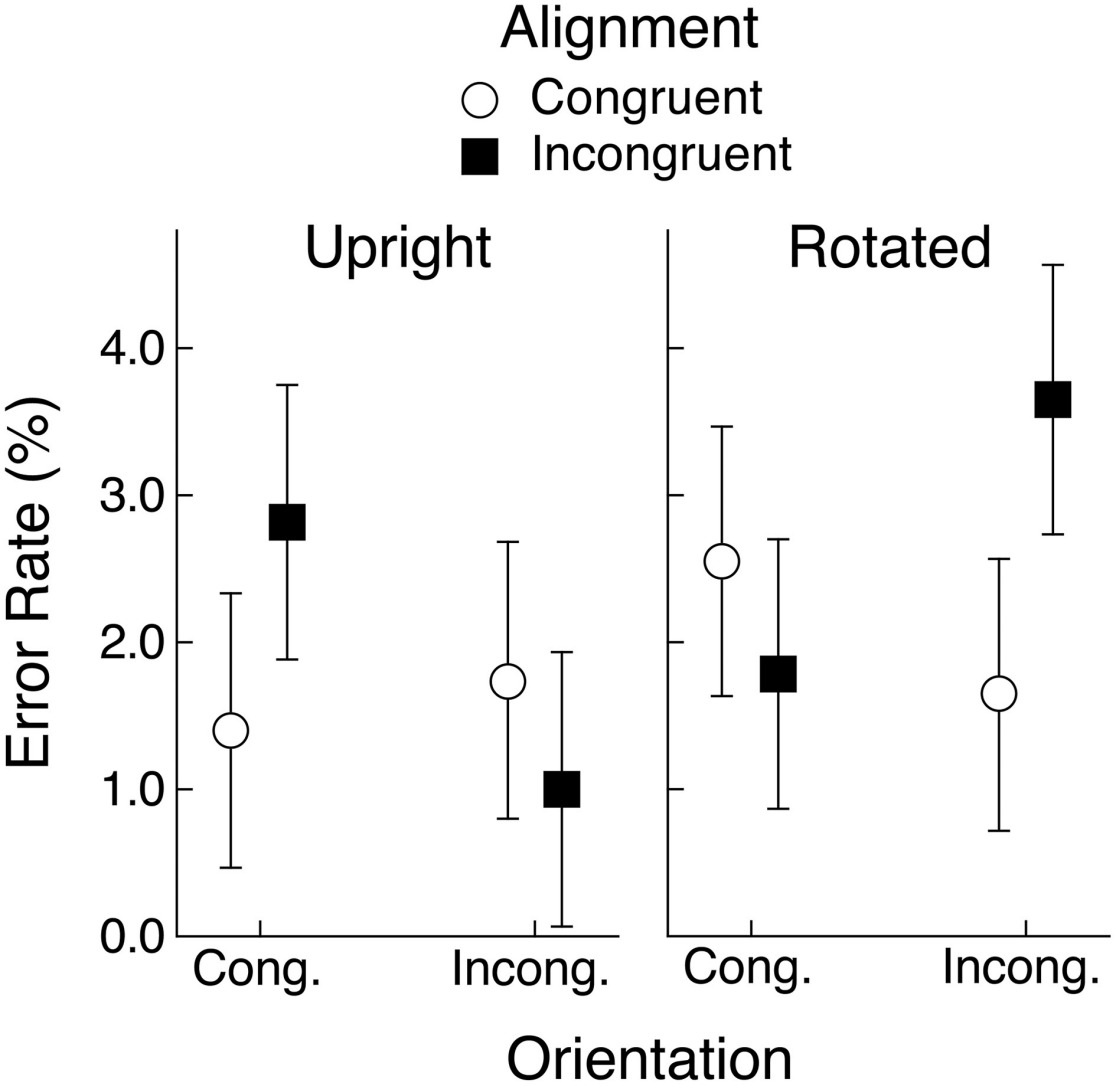
**Mean percent error in naming responses in Experiment 2 for upright and rotated views of objects having a canonical view**. Means are shown for the four conditions defined by congruency of alignment and orientation between the object's handle and the hand actions held in working memory. Error bars are 95% within-subject confidence intervals.

Mean error rates are shown in Figure 7 and it is apparent that congruency effects were similar to those obtained in the latency data. An ANOVA with object view and alignment and orientation congruency as repeated-measures factors found a significant effect of object view, with fewer errors on upright than on rotated objects (1.7 vs. 2.4%), although the effect was not supported by the Bayesian analysis, *F*_(1, 29)_ = 6.54, *MSE* = 4.09, *p* < 0.05, *BF* = 1.1. There was also a significant three-way interaction, *F*_(1, 29)_ = 8.18, *MSE* = 11.12, *p* < 0.01, *BF* = 160.1. No other effects were significant. Separate ANOVAs were computed for the upright and rotated conditions with alignment and orientation congruency as factors and the only significant effect from either analysis was the alignment by orientation congruency interaction for rotated objects, *F*_(1, 29)_ = 7.33, *MSE* = 7.88, *p* < 0.05, *BF* = 12.2. In general, the error data supported the pattern of congruency effects found in the response latency data.

### Aggregated data

**Figure 8:**
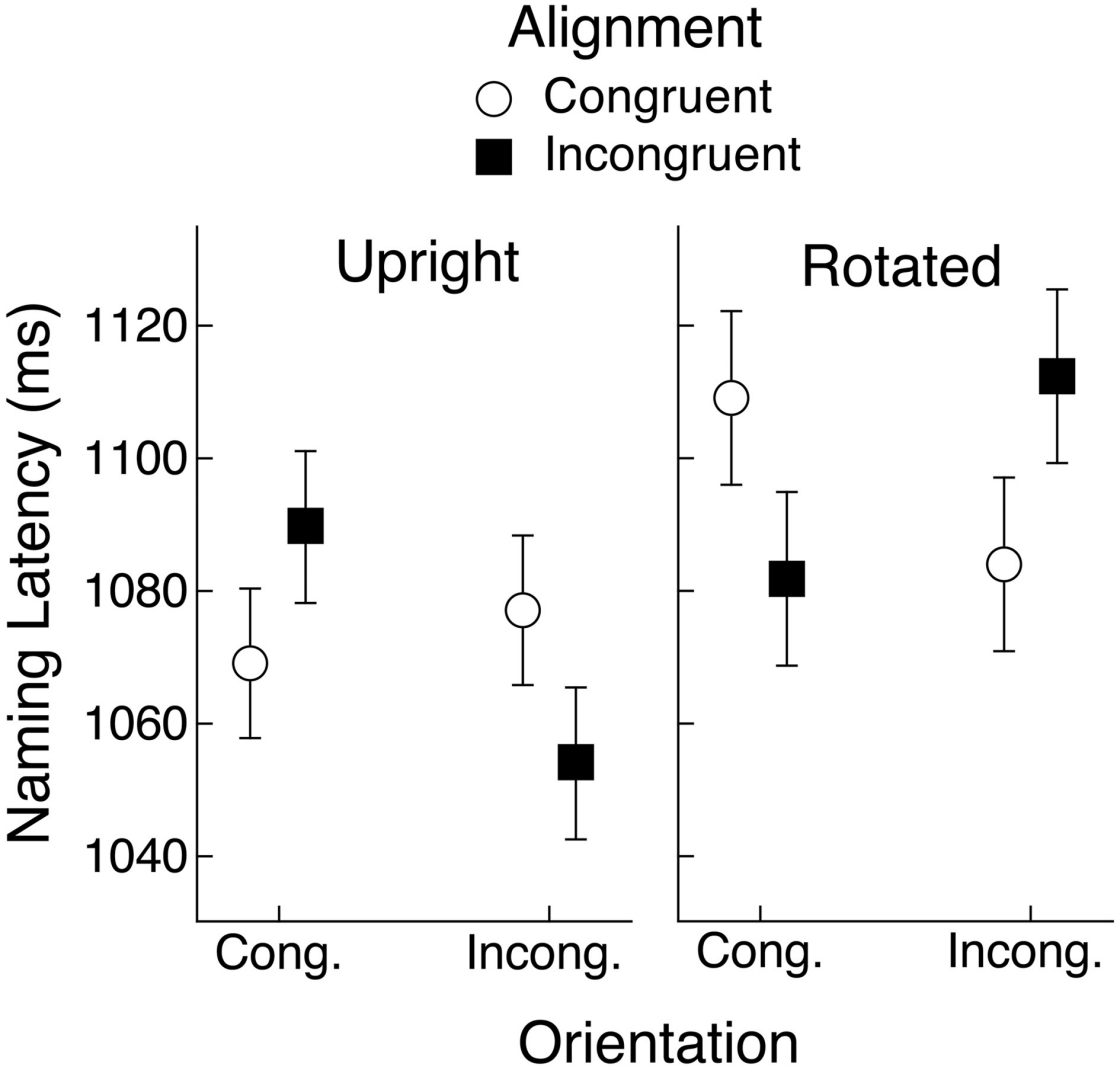
**Mean object naming latency averaged across Experiments 1 and 2 for upright and rotated views of objects having a canonical view**. Means are shown for the four conditions defined by congruency of alignment and orientation between the object's handle and the hand actions held in working memory. Error bars are 95% within-subject confidence intervals.

The data from Experiments 1 and 2 for objects that have a preferred view showed a tendency for alignment and orientation congruency effects to follow the partial overlap effect reported by Bub et al. (2013). These results did not, however, fully conform to that pattern. It is possible that by introducing the rotation manipulation we either reduced statistical power relative to the Bub et al. study, or perhaps even modulated the partial overlap effect. With these possibilities in mind, and given that both experiments produced a significant three-way interaction between object view and alignment and orientation congruency, we aggregated the data from the two experiments to yield a more reliable assessment of how well the pattern of that interaction conformed to the partial overlap effect in naming latency. The mean latencies are shown in Figure 8. The upright condition shows an approximation to the partial overlap effect, although the orientation congruency effect was weak under congruent alignment, as was seen within each of the two experiments separately. The rotated condition, however, showed a very clear replication of the partial overlap effect, assuming that object view was encoded according to the object's canonical, rather than its depicted view.

**Figure 9:**
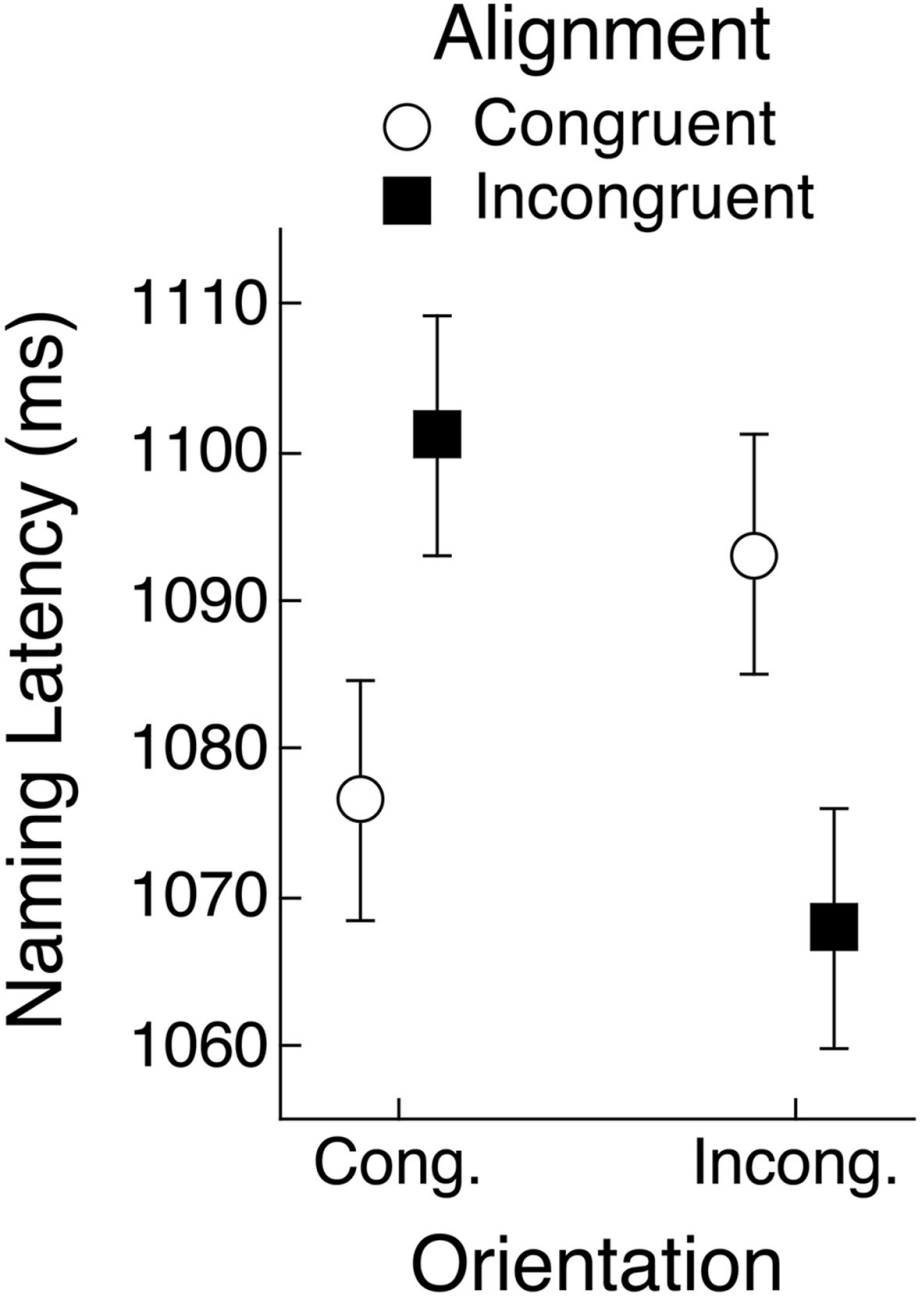
**Mean object naming latency averaged across Experiments 1 and 2 as a function of orientation and alignment congruency**. Orientation congruency was recoded for rotated objects to match the objects' canonical rather than depicted views and means are collapsed across the rotation manipulation. Error bars are 95% within-subject confidence intervals.

An ANOVA that pooled the latency data from both experiments and that included object view and alignment and orientation congruency as repeated-measures factors showed that naming responses were faster when the objects were upright (1072 vs. 1097 ms), *F*_(1, 59)_ = 20.05, *MSE* = 3554, *p* < 0.01, *BF* > 1000. The three-way interaction was also significant, *F*_(1, 59)_ = 36.47, *MSE* = 2035, *p* < 0.01, *BF* > 1000. No other effects were significant. Separate ANOVAs for each object view condition found no main effects but confirmed that the alignment congruency by orientation congruency interaction was significant for upright and for rotated objects (*p*s < 0.01, *BF*s > 100). These two-way interactions, of course, took opposite forms, suggesting that subjects had encoded rotated objects in their canonical view. Indeed, when we recoded orientation congruency for rotated objects so that it was defined by the objects' canonical rather than depicted view, the resulting ANOVA that included object view, alignment congruency, and orientation congruency, indicated that the significant alignment by orientation interaction (*F*- and *BF*-values were the same as reported above) was not significantly different for the two rotation conditions (*F* < 1, *BF* < 1). Mean naming latency as a function of alignment and orientation, collapsing across upright and rotated objects (with orientation in the latter case coded for the canonical rather than the depicted view) is shown in Figure 9. This pattern of means shows clear evidence for the partial overlap effect.

## General discussion

It has been widely established that action representations are automatically evoked by manipulable objects, even when such objects are passively viewed. This article concerns the possible contribution, if any, of these representations to perception. We developed a methodology that allows us to analyze the constituents of action invoked when participants engaged in the speeded naming of manipulable objects (see also Bub et al., 2013). Our approach is built on a procedure that previously has been used to examine how motor features in working memory adversely affect a subsequent perceptual or motor task demanding integration of these selfsame features (see Stoet and Hommel, 2002 for an overview). Performance suffers when only one of the features of the planned action overlaps with the sensory-motor features of the target event (e.g., Hommel, 2004; Fournier et al., 2013).

The pattern of interference effects has received the following interpretation. Assume that the contents of working memory include the motor features X and Z bound together into an action plan. Identifying the target object requires features X and Y. Feature X, activated by the perceived target object, primes the same feature held in working memory, leading to its automatic retrieval. However, retrieval of X also brings with it the bound feature Z. The feature Z now competes with feature Y, disrupting the ability to integrate Y with X as part of the representation of the perceptual target. In contrast, no such interference will occur for objects sharing both or neither of the features constituting the planned action.

Bub et al. (2013) examined the disruptive impact of action features in working memory on the ability to identify manipulable objects. We documented the same partial repetition cost previously obtained in numerous other studies relying on abstract symbols as targets and arbitrary actions as responses. Speeded naming was delayed and less accurate if the target object shared one of the action features in working memory; for example, if the contents of working memory involved the left hand and the palm oriented vertically, then performance was affected for a target object like a beer mug with the handle oriented to the right (wrist orientation matches the feature in working memory but not hand alignment) or a frying pan with the handle oriented to the left (hand alignment matches but not wrist orientation). Performance was faster and more accurate when the target object shared either both (e.g., a beer mug oriented with the handle on the left) or neither (e.g., a frying pan with the handle on the right) of the motor features in working memory.

The approach we have developed allows us to go well beyond previous demonstrations that secondary tasks involving some kind of action selectively disrupt the classification of manipulable objects (Witt et al., 2010; Yee et al., 2013). Our interest concerns the computational role of particular motor features in the identification of visual objects. In the present article we wished to determine whether these features correspond to actions triggered by the depicted form of the object or on a more abstract representation of the object's canonical form. The question is important because to identify an object requires that spatiotemporal information representing the particular token form of an object be mapped onto a more general description in long-term memory reflecting an object type. Accordingly, the role of action representations cannot be separated from the dynamic interplay between token and type descriptions activated during object classification.

## Perceiving rotated objects

We applied the method developed by Bub et al. (2013) to determine the nature of the action representations evoked by objects rotated 90° from their canonical view as well as by objects displayed in their typical upright view. The token or depicted view of a rotated beer mug affords a closed grasp with the wrist oriented horizontally. The object type, based on its canonical form, demands a closed grasp with the wrist oriented vertically. Which of these motor features—wrist horizontal or vertical—is evoked when subjects name the rotated beer mug? The nature of the partial repetition cost unambiguously indicates that a closed grasp triggered when naming a handled object always conforms to the wrist orientation associated with its typical upright view, even when the object has been rotated 90°. For example, actions in working memory incorporating a vertical wrist orientation interfered with the ability to name a rotated beer mug, despite the fact that the depicted view automatically triggers a horizontal grasp (Masson et al., 2011). This striking outcome allows us to infer that one component—wrist orientation—of the action representation consulted in naming a handled object is based on its canonical rather than its depicted form.

The object's depicted form, however, also exerts an influence on naming performance. The motor feature associated with a left or right handed grasp depends on the location of the opening or mouth of an object like a beer mug; the rotated form with the opening on the left affords a right- rather than a left-handed grasp if the object is returned to its upright (canonical) position. The partial repetition cost induced by the feature left/right hand is thus contingent on the depicted or token form of the object in relation to its canonical form. We access the upright description of beer mug when naming its rotated form but this representation includes a left- or right-handed grasp contingent on the object's initial view. A horizontal beer mug with the opening on the left translates into a beer mug with the handle on the right if rotated by 90° into an upright position, generating a right-handed, vertically oriented closed grasp. This action representation plays a role not only in the identification of an upright beer mug (handle on the right), but also in the identification of a horizontal beer mug affording the same grasp when rotated into an upright position.

## On the role of motor features in object identification

A standard result, also observed in the present article, is that naming is slower and/or less accurate for images of objects rotated in the plane than images of upright objects (Jolicoeur, 1985, 1988; Maki, 1986; Jolicoeur and Milliken, 1989; McMullen and Jolicoeur, 1990; McMullen et al., 1995; Murray, 1995, 1997). Convincing evidence has accumulated that the effect of rotation on performance occurs at a fairly late processing stage, and that an object's identity can be established independently of its view or token form. For example, neuropsychological cases have been documented who show accurate recognition of objects presented in different orientations, but severe impairment in assessing their orientation (Turnbull et al., 1995, 1997; Karnath et al., 2000; Harris et al., 2001). Thus, confronted with the image of an inverted dog, such patients, after identifying the animal, may contend that the dog's depicted view is canonical (upright).

Behavioral evidence confirms that establishing an object type or identity does not depend on the orientation of the token or depicted form. For example, Harris et al. (2008) briefly presented masked objects as primes followed by upright objects which were to be named as quickly as possible. The primes were displayed at varying degrees of rotation in the plane from an upright position. The magnitude of the priming effect did not depend on prime orientation, even though naming the same objects was systematically delayed as their orientation deviated from upright (see also Harris and Dux, 2005; Cheung and Bar, 2014, for additional evidence).

Although object identity can be determined independently of orientation, an object's orientation is an important aspect of its episodic representation. Chun (1997) has argued that an object's identity (i.e., its type) must be bound to a spatio-temporal representation (the token form of an object) to enable overt report (see also Harris and Dux, 2005). We conjecture that motor features driven by the depicted form of an object facilitate the binding of type-token descriptions. An upright beer mug with the handle on the left evokes a left-handed, vertical closed grasp, and the motor constituents of this action representation are part of the description that enable individuation of the object for conscious report. In effect, we identify the object as a “left-handed beer mug” because we integrate the object type (beer mug) with a particular token form yielding a left-handed grasp.

The depicted form of a beer mug displayed horizontally with the opening on the right evokes an action that begins with a horizontal left-handed closed grasp and ends with a vertical grasp. This action reflects the dynamic unfolding of a goal-oriented motor representation; a proximal grasp followed by an end-state of the action commensurate with the object's upright position (Masson et al., 2011). A right-handed grasp is not strongly activated by the rotated beer mug, because the proximal action consistent with this particular orientation requires an awkward counterclockwise rotation of the wrist to produce an upright object, a movement at odds with the end-state comfort effect (Zhang and Rosenbaum, 2008).

It is of considerable interest that naming rotated objects implicates a motor feature that reflects the distal goal or end-state of an action plan triggered by the object's token form. For an upright beer mug, the vertical wrist posture is the same for the beginning and end state of the grasp. For a horizontally oriented beer mug, the vertical wrist posture corresponds to the end state of the action triggered by the depicted view, whereas the proximal action involves a horizontal grasp. Because it is a vertical grasp that contributes to naming both upright and rotated depictions of a beer mug, the evidence suggests that the motor representation is based on the distal rather than proximal actions associated with the target object.

## Implications for apraxia

We conclude by returning to a conundrum posed at the beginning of this article. What is the relationship between naming an object and the actions determined by its form and function? We have conjectured that motor features are recruited as part of the spatiotemporal description of an object enabling conscious report. Motor features should play an increasingly crucial role when it becomes difficult to maintain a distinct episodic representation for a given object type. Under certain conditions, for example, it is hard to identify both instances of a repeated object presented within a 500-ms time window (the well-known repetition-blindness effect). According to Kanwisher (1987), repetition blindness occurs when an abstract description of an object (a *type*) is not encoded as a distinct visual episode (a *token*) because of the spatiotemporal uncertainty created by rapid serial visual presentation.

Interestingly, Harris et al. (2012) have shown that repetition blindness does not occur when the repeated item in a visual stream is the depiction of a manipulable object. In fact, these authors report a repetition advantage for manipulable objects, in contrast to the usual repetition blindness they obtained for non-manipulable objects. As Harris and colleagues suggest, motor representations associated with manipulable objects may enhance our ability to individuate two instances of the same object type. This possibility gives rise to a prediction concerning the performance of apraxic patients that is worth testing. Such patients can name objects despite impairments in accessing the motor representations associated with their function. How, though, would the performance of apraxic cases differ from age-matched controls, if greater difficulty occurred in establishing the spatiotemporal description of an object for conscious report? Given impaired access to motor features that help individuate an object, there should be no enhancement of the ability to identify two repeated instances of a manipulable object under conditions of rapid serial visual presentation. Unlike neurologically intact participants, then, apraxic individuals should demonstrate repetition blindness for both manipulable and non-manipulable objects.

## Conflict of interest statement

The authors declare that the research was conducted in the absence of any commercial or financial relationships that could be construed as a potential conflict of interest.

## Footnotes

^1^We did not use the two postures that were types of power grasps that had been included in the posture set used by Bub et al. (2013), because they were deemed too similar to the grasp used to hold the target objects. We wished to avoid the possibility that a third attribute, grip posture, might be shared between the target objects and actions held in working memory. The power grasps were replaced by a pointing index finger posture.
