# Supplementary material for: Components of action representations evoked when identifying manipulable objects
Source: Front Hum Neurosci. 2015 Feb 6;9:42. doi: 10.3389/fnhum.2015.00042 (PMC4319390; doi:10.3389/fnhum.2015.00042)
Supplement: Supplementary file 1 [file DataSheet1.DOCX]

**APPENDIX**

Names of Objects Used in Experiments 1 and 2

Acanonical Horizontal handle Vertical handle

flashlight frying pan beer mug

hairbrush iron coffee mug

hammer kettle garden sprayer

hatchet knife measuring cup

saw pizza cutter megaphone

sickle sauce pan pitcher

toothbrush strainer tea pot

wrench vaccum water pistol
